# Measurement Invariance of Negative Affect in Ambulatory Assessments of Young-Old and Old-Old Adults

**DOI:** 10.1093/geroni/igab046.2477

**Published:** 2021-12-17

**Authors:** Oliver Schilling, Anna Lücke, Martin Katzorreck, Ute Kunzmann, Denis Gerstorf

**Affiliations:** 1 University of Heidelberg, Heidelberg, Baden-Wurttemberg, Germany; 2 Heidelberg University, Heidelberg, Baden-Wurttemberg, Germany; 3 University of Leipzig, Leipzig, Sachsen, Germany; 4 Leipzig University, Leipzig, Sachsen, Germany; 5 Humboldt University of Berlin, Humboldt University of Berlin, Brandenburg, Germany

## Abstract

Gero-psychological research increasingly considered intense longitudinal assessments of momentary affect to address affective aging. In particular, many studies employed negative emotion item lists for ambulatory assessments of negative affect. However, frequent self-reports on emotion items within short time intervals might change alertness towards and perception of one’s emotional experiences. From an item-response-theoretic point of view, this might impair the stability of item functioning in terms of item discrimination between levels of affectivity and item severity (difficulty). Thus, we examined measurement invariance of negative emotion items commonly used for ambulatory assessments of negative affect. Ambulatory assessments from the EMIL study, obtained over seven consecutive days at six occasions per day from 123 young-old (aged 66-69) and 47 old-old (86-89) adults, were analyzed. Respondents self-reported on 13 negative emotion items, using a 0-100 slider to express the degree to which they felt the respective emotion. We ran multilevel structural equation models with Bayes estimation to analyze variability of negative affect factor loadings, item intercepts, and measurement error variances across repeated measures, thus checking for metric, scalar, and strict factorial invariance. For all sets of parameters, the findings do not strongly support measurement invariance, but point at partial invariance for item subsets. Taking on literature suggesting that criteria for invariance testing should not be too restrictive to meet pragmatic measurement equivalence requirements, further analyses and our conclusions focus on strategies that might allow for acceptable degrees of differential item functioning, enabling reliable analyses of intra-individual short-term variability in negative affect.

